# Attitudes and perceptions of medical doctors towards the local health system: a questionnaire survey in Ecuador

**DOI:** 10.1186/s12913-019-4211-1

**Published:** 2019-06-07

**Authors:** Esteban Ortiz-Prado, Marta Fors, Aquiles R. Henriquez-Trujillo, Gabriel H. Cevallos-Sierra, Alejandra Barreto-Grimaldos, Katherine Simbaña-Rivera, Lenin Gomez-Barreno, Eduardo Vasconez, Alex Lister

**Affiliations:** 1 One Health Research Group, Faculty of Medicine, Universidad de las Americas, Quito, Ecuador Calle de los Colimes y Avenida De los Granados, 170137 Quito, Ecuador; 2 Public Health Program, University of Southampton, Southampton, England

**Keywords:** Attitudes, Perceptions, Physicians, Health system, Medical doctors, Ecuador, Questionnaire

## Abstract

**Background:**

Health systems worldwide rely on health professionals to deliver services and provide framework structures. Considering their opinions about their work environment, the public policies that regulate their work and the deficiencies of the health care system are key aspects of the governance within the system. The aim of this study was to assess the perceptions of Ecuadorian physicians about several aspects of the performance of the health delivery and monitoring systems locally.

**Methods:**

A cross-sectional survey was performed in a group of physicians in Ecuador during 2017 using a self-selection sampling strategy. The participants were contacted by telephone, direct email or in person and asked to complete the online survey which contained 47 questions.

**Results:**

A total of 607 full responses were received from physicians, where 68.6% of those had graduated within the last 17 years. 46.4% of respondents were medical specialists, 23.1% general practitioners, 10.0% rural health practitioners, 9.5% sub-specialists, 5.9% were formally enrolled in a specialty program and 5.1% were researchers or administrative physicians. Data analysis of the answers showed that approximately 62% of physician respondents in the study found their current workload at the time of the survey was unmanageable, the most common complaints about the Public Health system in the study being the amount of daily paperwork (78.4%), followed by a perceived lack of vision of the health authorities (60.1%) and the resource limitations within the public hospitals (53.5%). Additionally, 71.8% of respondents referred to limitations of the National Essential Medicines chart- especially on the availability of some drugs- and 57.5% of the respondents reported concerns about the quality of medicines available for treatment.

**Conclusions:**

The data provide basic inputs for health authorities regarding the functioning of the health system in Ecuador. Health professionals’ concerns can be a valuable resource for monitoring and improving health system performance: however, there is a perceived sense of disconnection between the governance or management and the service delivery arms of the healthcare system in Ecuador. Whilst not representative of the entirety of the population of doctors, the study does give insight into where improvements to the health system might be made.

**Electronic supplementary material:**

The online version of this article (10.1186/s12913-019-4211-1) contains supplementary material, which is available to authorized users.

## Background

Health professionals are the backbone of health systems worldwide. The provision of quality care depends on the availability of a sufficient number of competent, committed and motivated health professionals working in a system with sufficient resources [1, 2]. The World Health Organization (WHO) has stated that an efficient health system consists of all organizations, people and actions whose primary goal is to promote, restore and maintain good health [3–5].

An adequate number of properly trained health professionals in the workforce are needed in order to achieve service goals [6]. Worldwide, health care providers are constantly exposed to a number of different service delivery challenges including high levels of stress, long working days with fewer adequate break times, crumbling or unfit infrastructure, limitations within their practices and overcrowded health services with waiting times always on the increase [7–9]. Although these problems vary from country to country, most of the health systems struggle in some degree with administrative and political issues, regardless of the income level or the standard of living of their citizens [10, 11].

Taking into account the opinion of health professionals about their work environment, the public policies that regulate their work and the deficiencies of the system from the inside is a key aspect of the governance within a health system [12–14]. As part of the ‘Strategy on Human Resources for Universal Access to Health and Universal Healthcare Coverage’ approved by the 29th Pan American Sanitary Conference on 2017, it is necessary to research the interests, motivations and required working conditions for health personnel in underserved areas in order to attract and retain human resources in such areas [15].

In Ecuador, decisions on public health issues usually respond to the different political views of the current government, leaving aside valuable information that could improve the functioning of the Ecuadorian health system [16, 17].

The health system of Ecuador it’s a mixture of a Bismarck and Beveridge model where the public government fund providers meet the privately own services and medical insurances [18, 19]. The public sector will cover more than 80% of the local population, including the uninsured (Minister of Public Health [MoPH]) and the public pensioners (Ecuadorian Institute of Social Security [IESS], Social Security Institute of the Armed Forces [ISSFA], the Social Security Institute of the National Police [ISSPOL]) [18], while the remaining ~ 20% will be covered by the private for-profit and non-profit organizations of civil society, including those with access to privately own medical insurances [17, 20].

Understanding the dynamics of this complex system from the perspective of a group of medical doctors (service providers) rather than from the perspective of patients (service users) will afford important insights into the challenges experienced- and attitudes towards- their health system [12, 21, 22].

### Objectives

The objective of this study was to gather information about the attitudes and perceptions of Ecuadorian physicians towards their workplace environment and the performance of the Ecuadorian National Health System.

## Methods

### Study design

A cross-sectional survey was conducted using an online questionnaire.

### Setting

The online questionnaire was implemented in the 24 provinces of Ecuador within the main public and private hospitals and medical centers in Loja, Quito, Guayaquil, Cuenca, and Machala. The online questionnaire was available for completion during the months of October, November and December 2017.

### Participants

The study demographic consisted of male and female medical doctors enlisted within the national registry for private providers (national level), the Provincial College of Physicians (provincial level) and the list of practitioners from main hospitals within five cities in Ecuador (see ‘Setting’ above). Study contributors were eligible to participate if they had graduated with a medical degree and were legally able to practice medicine in Ecuador. Consent from the participants was gained at the start of the questionnaire with an explanation of the aim of the study. Participants could continue to the full questionnaire only after consent was gained by agreeing (by electronically ticking) a ‘Terms and Conditions’ and ‘Agreement of Participation’ Assent Form.

### Data measurement and questionnaire

Prior to the study, a series of informal interviews were conducted with 20 experienced physicians and researchers from Ecuador, identified through a non-probabilistic purposive sampling exercise. This section of the project was carried out to identify key issues that may affect the relationship of healthcare workers with the health system in Ecuador. After the interviews, the final questionnaire was sent to those professionals previously recruited. After 2 months of proofreading the questionnaire, organizing the questions by sections and editing some of the questions that arose from the pilotage, a 47-item questionnaire, written in Spanish, was created entirely for the purpose of this project. An English-language translation version of the questionnaire was available as Additional file 1.

The research questionnaire had three sections:

- Section 1 explored demographic variables such as sex, age, university of graduation, postgraduate training, current place of work and workload;
- Section 2 measured the perception about issues related to the use of specific prescription drugs;
- Section 3 measured the participants’ experience in dealing with the Ecuadorian health system within the private and/or public sectors.

Each section of the questionnaire included a set of items in which the respondents were asked to choose a predefined answer listed after a question or statement. In addition, there were questions that required a ‘yes’, ‘no’ or ‘not applicable’ response. Progression to the next set of questions was not possible before answers to all the preceding questions had been registered. The pilot phase had indicated that it would take approximately 15–25 min to complete the questionnaire.

The distribution of the questionnaire was carried out via the email addresses and the telephone numbers included in the database of medical doctors enlisted within the national registry for private providers, the Provincial College of Physicians and the Human Resources Departments of the main hospitals in Quito, Guayaquil, Cuenca, Machala, and Loja. The link with the questionnaire was sent to the entire cohort of medical doctors (4000 individuals) and also via the encrypted instant messaging application within their smartphones. A brief explanation of the purpose of the study and assurance of the confidentiality of the data on the body of the email was assured in all cases. All the questionnaires were anonymized, and no identifiable data was requested.

### Bias

Non-response bias could have occurred in this study; important responses from eligible physicians of a particular demographic or those too busy to respond would be lost and therefore not accounted for in the results. We expected that those individuals who were more likely to complete the questionnaire might also have greater willingness to provide their own insights based on their experiences, thus, we cannot rule out some selection bias in this study. Finally, some responses about doctors’ pay could have been altered if participants were not feeling comfortable about sharing their monthly wages or wanted to inflate their true pay for unknown reasons.

### Study size

The sample size was calculated based on the estimation of the number of medical doctors in Ecuador according to the World Bank dataset on the number of physicians per 1000 population. This was calculated at 6710 doctors for the population of Ecuador in 2017 (16,776,977 inhabitants) [23–25]. Sample size calculation was carried out using a 99% confidence level with a margin of error of 5% and a response distribution set at 50%. The computed results suggested that a minimum of 603 subjects are needed to complete the questionnaire in order to achieve significance.

We collected 4000 email addresses and telephone numbers for the purpose of the study to which all were given an invitation to participate. At the end of the 3 months period, we had received 607 fully completed responses, reaching the minimum required quota for significance.

### Statistical analysis

Descriptive and inferential analysis was conducted using the software IBM SPSS Statistics for Windows Version 24.0. The results of each item in the questionnaire were reported as men and women in percentage and absolute frequencies with no further intersex variability analysis. The Chi-Square test was used to test the significance of association between numeric and nominal variables. Independent t-test for mean differences was calculated. Statistical significance was accepted at *p* < 0.05. Confidence intervals at 95% from means and proportions were also computed.

### Reliability and validation

Reliability was examined using a test-retest questionnaire using the final version of the survey. Since this questionnaire was created only for the purpose of this project, we tested within the cohort of experts previously selected for the informal interviews. Validation, on the other hand, was more challenging since this type of study had never been carried out in Ecuador or the region prior: thus we validated with previously ratified instruments measuring similar constructs in high-income countries [14].

## Results

The 4000 invitations to participate were distributed, via email by the researchers, to the defined population of physicians. Additional methods of distribution included using collaborators or through the publication of an online promotional article on social media. By the end of the data collection phase, 607 questionnaires were fully completed while 90 were closed with incomplete information- these surveys were not used in data analysis. The response rate of the survey was 17%; 59.9% of the respondents were male [95% CI:0.54–0.62] and 41.1% [95% CI: 0.37–0.45] were female. Most females in the sample were younger than men, averaging between 31 and 40 years old for females compared to 41–50 years for men.

### Professional qualities and experience

The majority of responses (68.6% of those used) were received from physicians who had graduated from medical school in the last 17 years. Of this percentile, 46.4% of respondents were medical specialists, 23.1% general practitioners, 10.0% rural health practitioners, 9.5% sub-specialists, 5.9% were formally enrolled in a specialty program and 5.1% were researchers or administrative physicians (Master of Public Health, Master of Science, Epidemiologists and PhDs in health-related fields).

Medical specialization is more frequent in males than in females, and those classified as ‘clinical’ were the most frequent specialization. The majority (93.4%) of doctors obtained their medical qualifications from local (Ecuadorian) universities. Responses were obtained from a range of regions, cities, and cantons in Ecuador, although the most represented cities were Quito, Guayaquil, Cuenca, Machala, and Loja. The majority of the participants (59%) work within the highland region, followed by the coastal region (36%) and the Amazon region (4%). Respondents came from publicly funded institutions (56%), private (28%) and others (16%). Table 1 displays the demographics of the participants.

**Table 1:** Participants’ demographics

Characteristics	Men *n* = 358 (58.9%)		Women *n* = 249 (41.1%)		*p* value
	n	%	n	%	
Group of age					
21–30	77	21.5%	84	33.3%	< 0.001
31–40	126	35.2%	101	40.1%	
41–50	61	17.0%	34	13.5%	
> 50	94	26.3%	33	13.1%	
Total	358	100.0%	252	100.0%	
Medical specialties					
Clinical	151	42.2%	95	38.2%	0.010
Surgical	49	13.7%	10	4.0%	
Clinical-Surgical	31	8.7%	13	5.2%	
Diagnostics	14	3.9%	8	3.2%	
Without specialty	113	31.6%	123	49.4%	
Total	358	100.0%	249	100.0%	
Place of study					
Local	334	93.3%	233	93.6%	0.892
Foreign	24	6.7%	16	6.4%	
Total	358	100.0%	249	100.0%	
Type of University					
Public	233	21.5%	153	61%	0.01
Private	89	24.9%	75	30%	
Co-financed	12	3.4%	3	1%	
International	24	6.7%	18	7%	
Total	358	100.0%	249	100%	
Region of the country					
Coast	123	34.4%	68	27.3%	0.172
Mountain	203	56.7%	162	65.1%	
Amazonia	24	6.7%	12	4.8%	
Insular	8	2.2%	7	2.8%	
Total	358	100.0%	249	100.0%	
Main Practice setting					
Public (MoPH)	182	50.8%	141	56.6%	0.429
Private	94	26.3%	51	20.5%	
IESS (Social security)	66	18.4%	41	16.5%	
ISSFA (Army)	3	0.8%	4	1.6%	
ISSPOL (National Police)	4	1.1%	4	1.6%	
Not working	9	2.5%	8	3.2%	
Total	358	100.0%	249	100.0%	
Functions					
Academy	21	5.9%	14	5.6%	0.651
Medical care	329	91.9%	225	90.4%	
Administrative	5	1.4%	6	2.4%	
Other	3	0.8%	4	1.6%	
Total	358	100.0%	249	100.0%	

Table 2 shows results regarding workload according to gender. Most participants (69%) reported working between 8 and 12 h a day. The typical 8 h day shifts are achieved by 36.7% of men and 53.8% of women. Physicians reported that 48% of them have to work during the night at least once a month, representing 8% of the overall workload among men and 12% among women. Only 1% of the responders work in 20 days in, 8 days off format. The differences in working hours between men (9.3 h/day) and women (9.8 h/day) were assessed using an independent t-test for mean differences, the results were not statistically significant (0.58 [95% CI: 0.05–0.92]).

**Table 2:** Questions related to income, night shifts and perception of other colleagues

Q10: How many hours Per Day do you work?	Men		Women	
4 Hours/day	20	5.5%	0	0%
5 Hours/day	0	0.0%	1	0.4%
8 Hours/day	133	36.7%	134	53.8%
9 Hours/day	0	0.0%	17	6.8%
10 Hours/day	69	19.3%	45	18.1%
11 Hours/day	12	3.5%	9	3.6%
12 Hours/day	96	27.0%	28	11.2%
13/Hours/day	8	2.3%	5	2.0%
14 Hours/day	8	2.3%	2	0.8%
15 Hours/day	2	0.6%	1	0.4%
16 Hours/day	8	2.3%	3	1.2%
17 Hours/day	1	0.3%	0	0.0%
18 Hours/day	0	0.0%	1	0.4%
20 Hours/day	1	0.2%	3	1.2%
Total	358	100.0%	249	100.0%
Q11: Do you work Saturdays?				
Yes	172	48.0%	96	38.6%
No	186	52.0%	153	61.4%
Total	358	100.0%	249	100.0%
Q12: Do you work night shifts?				
Yes	172	48.0%	96	38.6%
No	186	52.0%	153	61.4%
	358	100.0%	249	100.0%
Q13: Monthly Income				
Specialized	$ 3872.0		$ 3309.68	
General Practitioner	$ 1698.0		$ 1782.43	
Resident	$ 1762.0		$ 1447.62	
Rural MD	$ 1497.0		$ 1351.52	
Other	$ 3106.0		$ 2562.00	
Academy	$ 3394.0		$ -	
Fellowship	$ 5020.0		$ -	
Q21: What is the % of Doctors in Ecuador doing a bad job				
< 10%	73	20.4%	54	21.7%
10–20%	70	19.6%	58	23.3%
30%	78	21.8%	58	23.3%
40%	48	13.4%	31	12.4%
50%	40	11.2%	25	10.0%
60%	18	5.0%	11	4.4%
70%	21	5.9%	10	4.0%
80%	8	2.2%	1	0.4%
> 80%	2	0.6%	1	0.4%
Total	358	100.0%	249	100.0%

Information about income according to gender and type of specialization is presented in Table 2. Differences between male and female doctors are seen in all types of health professionals. Males in most sections earned more than females except for those in general practice. In the overall average, male doctors earn more money on a monthly basis than female doctors ($2907 vs $1493 US). When comparing females versus males in terms of three categorical monthly salary ranges (<$3000, $3000–$6000 and > $6000 US) the Chi-square statistic was 6.4499 with a *p*-value of 0.039, showing that the differences are statistically significant.

**Fig. 1:**
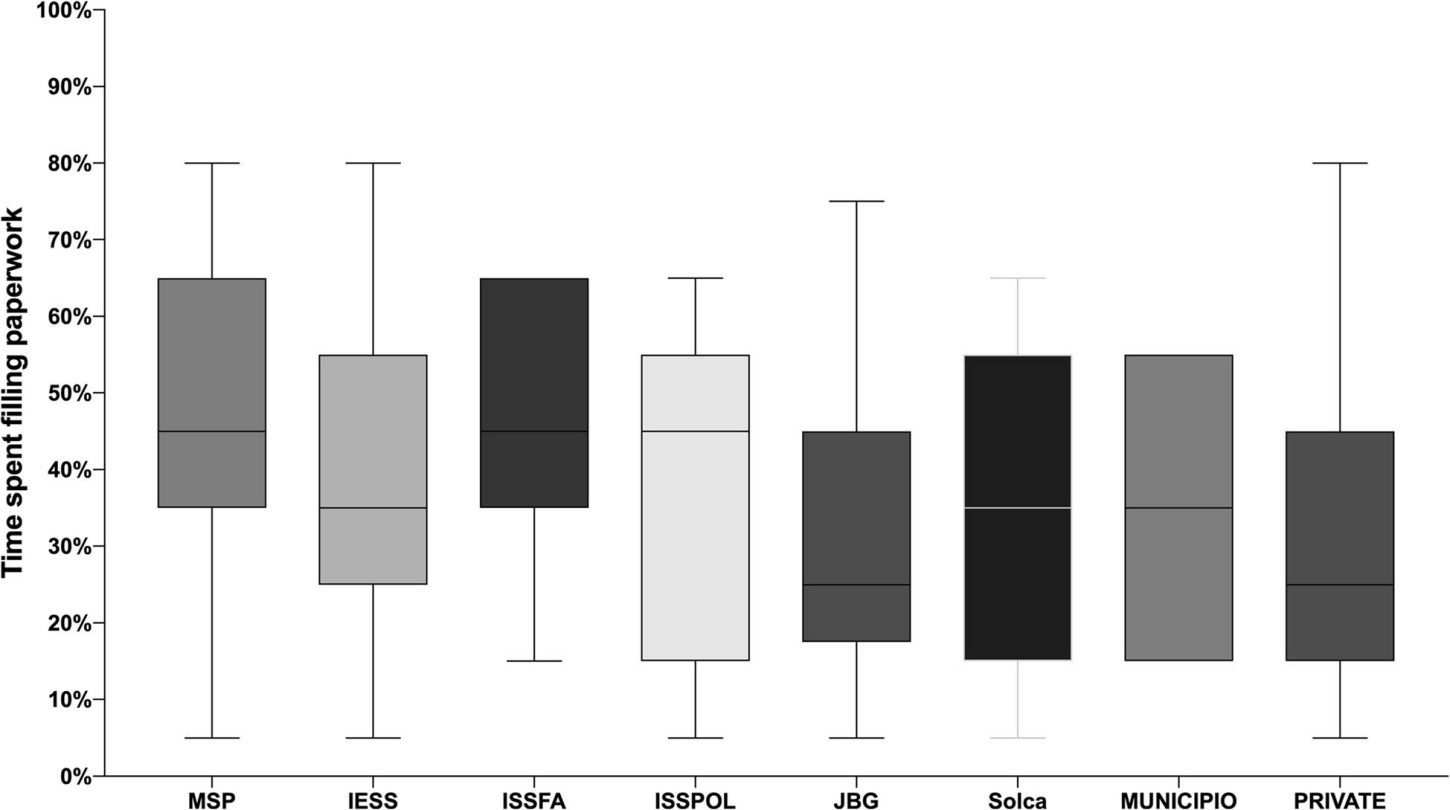
Total amount of time dedicated to fill paperwork by institution. MSP: Hospital from the Minister of Health, IESS: Hospital from the Public pensioners funds belonging to the Ecuadorian Institute of Social Security, ISSFA: Hospital from the Social Security Institute of the Armed Forces, ISSPOL: Hospital from the Social Security Institute of the National Police, JBG: Hospital from the Guayaquil Charity Board, SOLCA: Hospital that offered oncological care only, Municipalities: Those clinical centers that are part of the cities’ local governments, Private: Hospitals that are fully funded by private funds

Figure 1 reports the measured duration of time that is devoted by health professionals in completing administrative forms related to their work. Physicians working at public facilities were found to devote more time doing the paperwork than those working in other care settings.

**Fig. 2:**
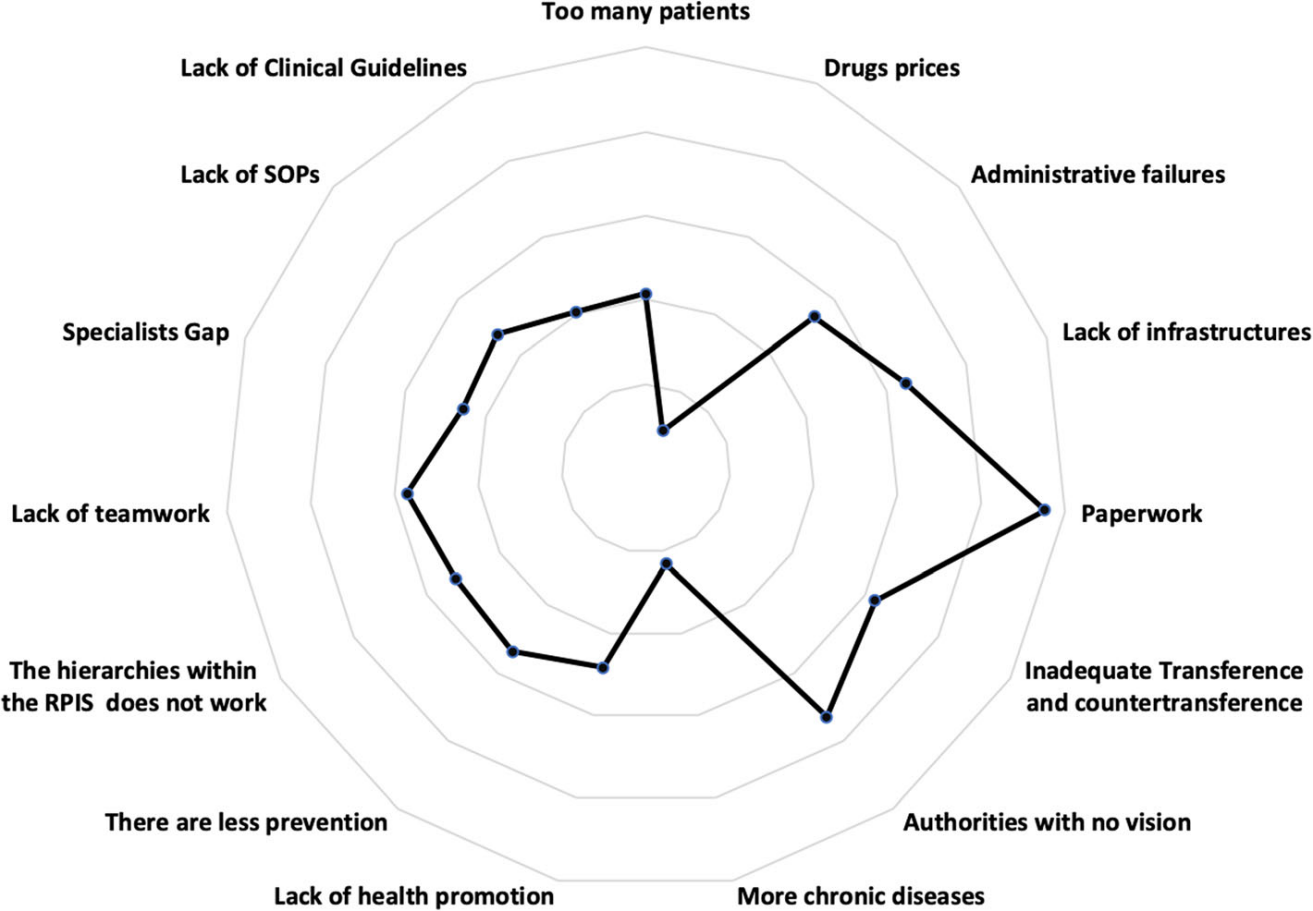
Radar graphic about the main problems faced by the local health system

Table 3 shows information on key perceptions regarding the organization of the Ecuadorian health system. We found that most physicians (62%) disagree with the workload of 8 h a day for specialists in the public sector. 48% would be willing to work 4 h a day for a proportional wage and 36% would like to earn as much as fulltime position (8 h) despite working only 4 h. For 8% of respondents working in the public sector would never be a career option and the last 8% did not respond.

**Table 3:** Questions about health care organization in Ecuador

Questions	No.	%
Q.14 Do you agree with the work schedule of 8 h a day for specialists?		
Yes	233	38.0
No	337	62.0
Q. 15 Should a 4-h position be opened in a public hospital; would you be willing to work there?		
Yes, I would work for a proportional part-time wage (4 h /day)	229	48.0
Yes, I would work 4 h, but I want to earn much as a full-time wage (8 h/day)	227	36.0
No, I would never work in the public sector	52	8.0
Other	44	7.0
Q.16 In your opinion, what are the main problems of the Public Health Sector? (one or more answers can be chosen)		
There is too much paperwork	476	78.4
There is a poor vision from health authorities	465	60.1
There are deficiencies regarding the hospital infrastructure	325	53.5
There is not a team work philosophy	285	46.9
There is a predominance of the curative approach rather than a preventive approach	269	44.3
Administrative personnel do a poor management job	269	44.3
The hierarchical structure within the Public Sector does not work properly	261	43.0
Health promotion needs improvement	242	39.8
Lack of clear processes and procedures to deal with difficult situations	237	39.0
The processes of referral of patients is mismanaged	214	35.2
There are too many patients	207	34.1
Protocols and clinical guidelines are non-existent. This difficult standardization of treatments	203	33.4
Other	123	20.2
There is an increase in chronic and noncommunicable diseases	116	19.1
Medical costs are too high	49	8.0

### Perceptions

The survey inquired about participant’s perceptions about the most prominent problems or issues within the Ecuadorian public health sector. Participants could choose more than one option. In Table 3 we present the percentages of responses related to the questions answered. It must be stated that since participants could choose more than one answer, the total responses exceed the number of participants. Interestingly, the most pressing issues for the public sector in health perceived are that there is too much administration to do within the working day, (with a marked 78.4% consensus), that there is a poor vision from health authorities (60.1%) and that there are deficiencies regarding the hospital infrastructure (53.5%).

Figure 2 Most frequently stated opinions on the health service from physicians in Ecuador, relative to each other. Greater weighting is shown towards the amount of paperwork and the lack of vision of the authorities

Table 4 shows the results on the opinions of the public health system in the country. Most of the respondents knew what the Public Integrated Network of Healthcare Services (RPIS) was, but they were not aware of the contents of the National Code of Health (COES) under debate in the National Assembly which regulates the National Health System. Most (79%) agree on the necessity to implement regulations to diminish malpractice in the country. 71.8% of respondents refer to limitations of the National Essential Medicines chart, especially on the availability of some drugs. Over half of the physicians (55%) think that generic drugs available in the country are of poor quality. Approximately 29% of the participants were not willing to assist patients in the public health system at no cost.

**Table 4:** Opinions about the Public Health System in Ecuador

	Specialists (*n* = 282)	GP* (*n* = 141)	Rural GP (*n* = 62)	Others (*n* = 28)	PGY** (*n* = 36)	Fellows (*n* = 58)
Q. 18 Do you know what is the Integrated Networks of Healthcare Services (RPIS)?						
Yes	74%	79%	76%	82%	78%	81%
No	4%	3%	0%	0%	6%	11%
Not Sure	19%	13%	22%	11%	17%	8%
Vaguely	3%	5%	2%	7%	0%	0%
Q. 19 How much do you know about the National Code of Health (COES)						
Don’t know	2%	4%	2%	0%	3%	7%
Haven’t read it, just heard about it and I Know a little	28%	34%	32%	21%	25%	26%
Haven’t read it, just heard about it and I Don’t know nothing	10%	10%	11%	7%	11%	14%
Read it by parts and know part of it	51%	45%	50%	50%	61%	45%
Read it and I know it all	8%	7%	5%	21%	0%	9%
Q. 20 Do you think is necessary to have a medical malpractice law in Ecuador?						
Yes	79%	67%	73%	86%	61%	74%
No.	11%	23%	15%	7%	22%	13%
Other response	11%	10%	13%	7%	17%	13%
Q. 24 What is your perception of the usefulness of the National Chart of Basic Drugs in Ecuador (CNMB)						
Very useful	2.1%	4.1%	0.0%	7.7%	0.0%	3.0%
Useful	10.1%	14.5%	26.1%	15.4%	4.2%	2.0%
Unlikely to be useful	11.8%	9.5%	5.4%	23.1%	16.7%	13.1%
Limits access to medicines	34.2%	34.1%	32.6%	23.1%	33.3%	32.3%
Excludes Important medicines	28.0%	28.6%	28.3%	21.5%	37.5%	37.4%
Includes no recommended drugs	8.9%	5.0%	4.3%	6.2%	2.1%	8.1%
Improves the correct use of medicines	3.9%	3.6%	3.3%	3.1%	6.3%	4.0%
Unknow Information	1.0%	0.5%	0.0%	0.0%	0.0%	0.0%
Q. 25 What is your opinion about the generic medicines that are marketed in Ecuador?						
Same Quality as Brand Medicines	5%	5%	8%	9%	0%	8%
Most of them are good	33%	46%	47%	91%	100%	92%
Most of them are bad	63%	49%	45%	0%	0%	0%
Q. 33 Do you agree that all medical students after completing their degree have to take a qualification exam before practicing?						
Yes	72%	71%	93%	92%	80%	71%
No	28%	29%	7%	8%	20%	29%
Q. 34 Do you think that specialists and general practitioners should undertake a “re-certification” process every 5 years?						
Yes, with an exam	7.4%	9.9%	11.3%	3.6%	11.1%	5.2%
Yes, throughout CME	75.9%	70.2%	54.8%	75.0%	72.2%	75.9%
Other	2.5%	0.0%	3.2%	3.6%	2.8%	10.3%
No	14.2%	19.9%	30.6%	17.9%	13.9%	8.6%
Q. 35 How good or bad represented do you feel by the Ecuadorian Medical Federation (FME)						
Extremely Bad	18.1%	11.3%	14.5%	21.4%	13.9%	25.9%
Very Bad	11.0%	9.2%	3.2%	14.3%	2.8%	3.4%
Bad	14.9%	16.3%	22.6%	10.7%	30.6%	29.3%
Neither good/bad	34.0%	37.6%	33.9%	32.1%	44.4%	29.3%
Good	14.5%	18.4%	22.6%	17.9%	5.6%	5.2%
Very Good	5.0%	7.1%	1.6%	3.6%	2.8%	5.2%
Extremely good	2.5%	0.0%	1.6%	0.0%	0.0%	1.7%
Q. 36 In general, what do you think about the scientific quality level of Ecuadorian scientific societies?						
Extremely Bad	7.8%	0.6%	6.4%	10.6%	14.2%	5.2%
Very Bad	5.0%	16.6%	3.2%	3.5%	0.5%	5.2%
Bad	16.0%	14.6%	21.0%	7.1%	22.7%	22.4%
Neither good/bad	42.6%	35.0%	37.1%	38.9%	51.1%	43.1%
Good	18.4%	21.7%	27.4%	38.9%	8.5%	15.5%
Very Good	8.9%	10.8%	4.8%	0.8%	2.8%	8.6%
Extremely good	1.4%	0.6%	0.1%	0.1%	0.2%	0.1%
Q. 38 If you were asked, would you be willing to attend certain number of patients from the public health system at no charge at your private medical practice? for free in your private practice?						
1/week	11.3%	11.3%	8.1%	7.1%	16.7%	12.1%
2/week	14.2%	19.1%	11.3%	7.1%	5.6%	6.9%
3 /week	12.8%	19.1%	12.9%	10.7%	8.3%	17.2%
4/week	31.9%	34.8%	54.8%	50.0%	52.8%	34.5%
None	29.8%	15.6%	12.9%	25.0%	16.7%	29.3%

*GP General Practitioner, **PGY Post Graduate Physician

Table 5 highlights the types of drugs that medical staff have had difficulty in sourcing. The greatest difficulty was found in the availability of antineoplastic, antibiotics and antihypertensives.

**Table 5:** Which pharmaceutical products are harder to find in Ecuador

^a^Pharmacological groups	n	%	Example Drugs
Antineoplastic	49	20.1	Pertuzumab, Methotrexate
Antibiotics	43	17.6	Ertapenem, Ciprofloxacin
Antihypertensives	42	17.2	Losartan, Nifedipine, Methyldopa
Analgesics/anesthetics	30	12.3	Paracetamol, Metamizole
Hypoglycemic agents	21	8.6	Insulin, Metformin
Anti-inflammatory	13	5.3	Hydrocortisone, Betamethasone
Anticonvulsants	12	4.9	Carbamazepine
Antidiarrheals	11	4.5	Loperamide
Diuretics	8	3.3	Chlortalidone, Furosemide, Hydralazine
Hypolipidemic drug	8	3.3	Simvastatin, Gemfibrozil
Antiparasitic drugs	7	2.8	Meglumine antimoniate
Total	244	100.0%	

^a^Q 29 In your experience, which is or what are the most inaccessible pharmaceutical products in Ecuador (Choose up to five)

## Discussion

The results of this study can be useful to understand the complex reality of the healthcare workforce in Ecuador and can help to better plan some of the next steps to improve the Ecuadorian strategy for healthcare human resources. Ecuador has implemented several reforms aimed to strengthen a health care model based on primary care. The 2008 Ecuadorian Political Constitution gave the Ministry of Health (MoH) increased power to shape and reorganize the health system to achieve universal healthcare. Human Resources Management changed focus to be better aligned with a system that sought to consolidate an organization based on integrated networks of healthcare services [26]. These reforms resulted in an increase in the number of healthcare professionals within the public sector during the last decade [26]. This was due mainly to the implementation of the economic incentives to attract and retain qualified personnel in rural areas where they are much needed. According to MoH authorities, besides monetary compensation for geographical allocation, a better, more positive work environment was created with the availability of sufficient resources making it easier for healthcare professionals to move within the country [20].

Doctors’ attitudes towards their work environment reflect their perceptions about the organization and performance of the healthcare system and may influence their own behavior in practice. The majority of respondents were medical specialists working in public hospitals in large cities, which is congruent with the unequal distribution of health professionals in the country. Surprisingly, there were no family doctors in the respondents.

The most pressing issue regarding the Ecuadorian healthcare service, according to the respondents, was related to the amount of administrative paperwork facing professionals on a daily basis. The public healthcare service of Ecuador does not have a centralized computer information system meaning that administrative tasks such as appointments, drug procurement, referrals, record management, and statistical updates need to be entered manually, and healthcare providers must complete all orders and reports by hand in order to record their work.

All information is then transferred to spreadsheets by a third person in order to send them to regional or national Management Offices. In any case scenario, it is impossible to access local information directly or to make a follow-up inquiry for a case between providers. Ecuadorian regulations require the use of a unique and unified clinical history format by all professionals. This is the case even in places that have an electronic information system running in parallel, (such as highly specialized hospitals). Professionals, therefore, must do both tasks; registering information on the local computer system and also making the physical paper record and this is also essential for the interaction between sub-systems- when a patient is referred from a public service to the Social Insurance Information System as an example. All records and orders must be registered on paper in order to request payment from the public system, but they also need to be registered separately in the Social Insurance Information System.

The second biggest issue reported, a perceived poor vision from health authorities, can be interpreted as a general lack of understanding of the far-reaching objectives and proposed impacts of the reforms undertaken by the Public Health Ministry. In general, Ecuadorian physicians appeared frustrated with the lack of communication and feedback they receive from the health system institutions and administrators.

Most physicians report being dissatisfied with their work schedule. In Ecuador, prior to the reforms of the health system started by the government, specialists were able to work in 4-h shifts for a five-day working week- Monday to Friday. Such an arrangement allowed professionals with a specialism to work part-time in the public sector and continue practicing for private providers. This arrangement was considered to be a conflict of interest by the authorities, and therefore a rule was passed that forced public servants to fulfill 8 h of work in a working day in order to continue being employed within the public system. Some experienced physicians chose to renounce their public positions in favor of the private sector. Statistically, the survey showed that most physicians who expressed a preference, are not in favor of this schedule and that a group still voices their objections to the change.

The third most reported issue was regarding a perceived lack of hospital infrastructure. This issue is given added weight since Ecuador has increased its spending on infrastructure during the last 12 years. There have been a record number of new healthcare units and small hospitals, as well as improving and upgrading both the equipment and infrastructure in traditional, locally based hospitals. As a caveat, it is possible that the political polarization occurring in Ecuador during the last 4 years has contaminated the views of professionals against anything government- related, including public spending on health.

According to the majority of participants in the study, the drugs in the basic table are not sufficient to afford patients adequate treatment. The lack of medication and supplies due to the inadequate distribution of resources for the health sector directly affects the fulfillment of the doctor’s duty of care [27]. This is also the case of the perceived utility of the Essential Medicines Chart (EMC). Most physicians consider it ‘not very useful’. The EMC defines the essential medicines for the public healthcare system. Under this approach, public healthcare providers can only access medicines contained within; a thorough analysis of the pharmaceutical compounds contained on the EMC is performed by the National Health Council (CONASA) and a revised list is published every 2 years. The council has a focus on primary healthcare and intends to cover the majority of the diseases present in the country. The lack of utility perceived by the respondents probably has to do with the high number of specialists among respondents, as well as the lack of family doctors among the sample. A common complaint among specialists in public hospitals is that the EMC limits the availability of new and ‘modern’ drugs for their specialties. This is likely to be true since the EMC also limits the number of pharmaceutical compounds that can be purchased for the public system to those with the greatest and highest evidence of safety and benefit. New drugs usually lack this type of information.

Another interesting finding is the perception of the quality of generic drugs available in Ecuador. Just over half (55%) in the study think that this group of drugs are of poor quality, while 34% rated the majority of drugs as ‘good’. Only 10% of respondents had a perception that generics are as good as brand pharmaceuticals, differing from the results published elsewhere [22]. The number of physicians who railed against generic drugs could be influenced by the promotion techniques of the transnational pharmaceutical companies that others have shown [28].

The National Pharmaceuticals Regulatory Agency (NPRA) has exercised significant efforts to introduce pharmacovigilance within the healthcare providers from 2013 onwards, as part of the strategy to guarantee the quality of drugs purchased centrally for the public system. Up to now, this measure has produced mixed results. The NPRA has a project underway to obtain an international certification by the WHO as an internationally recognized agency but unfortunately, despite more than 5 years’ work, the agency has not been able to obtain it. The outcome is that as things currently stand Ecuador does not have an agency with an internationally recognized process to guarantee the quality of drugs purchased by the public centralized procurement system. Under these circumstances, the perception of physicians about the quality of generic drugs must be considered very seriously [22].

As we have seen, there seems to be a clear disconnection between the management and operative branches of healthcare in Ecuador. The reforms undertaken by the government have not been presented in their best light to healthcare personnel to obtain buy-in with the attendant change in attitudes and practices. A significantly important proportion of physicians in the sample seem to be desirous of returning to a part-time work schedule, even if this is not the best thing for the system.

It is also interesting to note that a significantly high proportion of physicians consider the governing bodies to have no vision or ability to lead the efforts to improve the system. This is particularly challenging when dealing with public health interventions that have been evidenced elsewhere to be efficacious in improving system performance overall and the provision of services generally. Examples of such interventions are those of central drug procurement, Essential Medicine Charts, and generic drugs- clearly good steps towards achieving universal healthcare but need to be better presented to the health workforce to obtain critical support.

Health care professionals, being the first point of contact with patients, or service users, are often blamed when the system is not working properly. Evaluations and quality surveys are constantly delivered to patients by local authorities and insurance companies- however, rarely do physicians have to provide any formal form of evaluation and provide feedback about their work environment [29–32]. Health workforce management systems have focused on evaluating their performance through systems that do not usually take into account the perceptions of professionals about their work environment, nor the overall quality of the health system [33–36]. The quality of services provided, including the effects of the workplace environment towards health professionals, have been usually assessed in high or middle-high income countries where the quality of services is well known [37–39]. On the other hand, low and middle-lower income countries are facing different issues, usually related to equal access to healthcare rather than the quality of those services.

Although this study does not represent entirely the reality of all healthcare workers in Ecuador, the findings present an accurate picture of trends in doctors’ opinions from them. It is recommended that a survey study with adequate representativeness of all healthcare workers should be undertaken at some time in the future. For the time being, the results from this study should certainly be of interest to Ecuadorean policy and decision makers, (particularly about the lack of adequate information among their healthcare workers)- this could help influence good health policy as well as compromise the achievement of long-term goals and governability if the root cause issues remain unaddressed.

### Limitations

This study has some limitations to consider. It was a descriptive and exploratory study without any method of weighting data to make adjustments for how confounding variables could have influenced responses. Additionally, due to the nature of the study (online 15–25 min long questionnaire), we did not design the sample to statistically represent the population of physicians of Ecuador and make rigid extrapolations, but to offer for the first time, in Ecuador, useful insights of the attitudes and perceptions of local physicians towards the health system.

Only doctors with internet availability, an active e-mail account and who publicly shared their phone number were eligible to participate; therefore, it must be accepted that a significant proportion of physicians and their opinions were missed in this analysis. Given that the average length of time required to complete the survey was around 15 to 25 min, (and there were no large deviations from this time) it is likely that those physicians with a higher workload, and therefore unwilling or unable to commit that time, were missed. The response rate of the survey was probably influenced by the internal decision to stop gathering data when the calculated sample size quota was achieved (603 fully completed responses). We are aware that this decision might exclude those participants who received the active survey’s link later than the rest but did not complete their responses within the 3 months period.

Non-response and selection bias cannot be ruled out, and due to the non-probabilistic nature of the sampling strategy, caution must be taken in conclusions.

## Conclusions

Physicians are an important group within the public health system of the country and face many challenges in improving the quality of medical care. The results of this survey show that there are important issues that need to be solved to address medical doctors’ day-to-day needs and their beliefs as to how the system might be better structured, resourced and governed. Educational programs tailored to providing physicians’ knowledge about the system are needed in the country.

The data provides the basic components for Health Authorities to start to take important decisions regarding the functioning of the system and to formulate ways to address the problems as set out in the survey: nevertheless, further research is needed for a proper study to inform policy.

The perceptions from physicians and the health authorities seem to be suffering from a disconnect between the Management and Operative/Service Delivery branches of healthcare in Ecuador in terms of the reality of the situation. The reforms already undertaken by the government have not been positively structured and presented to healthcare personnel in such a way as to gain buy-in, and has therefore failed to provide any motivation for addressing the deficiencies in knowledge, attitudes, and practices. A significant proportion of physicians in the sample stated their eager desire to return to a part-time work schedule, despite their acknowledging that this is not the best thing for the health delivery system overall. Although the results do not reflect the perceptions of the entirety of the workforce of doctors in Ecuador, it does give an insight into how some perceive their place of work.

## Additional file

Additional file 1: A Questionnaire Survey in Ecuador (Translated to English). Attitudes and Perceptions of Medical Doctors Towards the Local Health System Questionnaire. A 47 questions online Questionnaire. (DOCX 23 kb)

## Data Availability

The information from the entire dataset is available under an informal written request.

## Abbreviations

CEISH-UDLA: Human Research Ethics Committee of the Universidad de las Americas
COES: National Code of Health
COIP: Integral Penal Code
CONASA: National Health Council
EMC: Essential Medical Chart
IRB: Institutional Review Board
MoH: Ministry of Health
MoPH: Minister for Public Health
NPRA: National Pharmaceuticals Regulatory Agency
RPIS: Public Integrated Network of Healthcare Services
UDLA: Universidad de las Americas
WHO: World Health Organization
