# Supplementary material for: Attitudes and perceptions of medical doctors towards the local health system: a questionnaire survey in Ecuador
Source: BMC Health Serv Res. 2019 Jun 7;19:363. doi: 10.1186/s12913-019-4211-1 (PMC6556044; doi:10.1186/s12913-019-4211-1)
Supplement: Supplementary file 1 — A Questionnaire Survey in Ecuador (Translated to English). Attitudes and Perceptions of Medical Doctors Towards the Local Health System Questionnaire. A 47 questions online Questionnaire. (DOCX 23 kb) [file 12913_2019_4211_MOESM1_ESM.docx]

**Attitudes and Perceptions of Medical Doctors Towards the Local Health System: A Questionnaire Survey in Ecuador**

1. A. General Information: Sex
   a. Woman
   b. Man

B. General Information: Date of birth

_______________________________________

1. University of graduation
2. In what year did you obtain your title of General Physician? (only the year of graduation)

____________________________________________________

1. What is your level of academic education?
2. General Physician
3. Specialist
4. Postgraduate Resident
5. Rural Physician
6. Sub-specialist
7. Other
8. What qualifying specialty (medical specialty) or professional (master's or doctorate) do you have?
9. What is your current main work activity, your occupation or work within the health system?
   - 1. General Physician
     2. Specialist
     3. Postgraduate Resident
     4. Assistance Resident
     5. Rural Physician
     6. Sub-specialist
     7. Teacher / Professor
     8. Other
     9. None
10. Do you do any administrative, normative or technical function within the national health system? Indicate the corresponding *** If you are not sure, indicate Not Apply
    - 1. Yes, I do other administrative functions
      2. Yes, I work in the area of epidemiology, public health, surveillance, data, among others.
      3. Not apply
11. The place where you work belongs to which of the following institutions (you can choose more than one)
    - 1. Ministry of Public Health
      2. IESS
      3. ISSFA
      4. Private (Hospital or Clinic)
      5. Guayaquil Charity Board
      6. SOLCA
      7. Private Medical Office
      8. No longer Exercise
      9. Academy (Teaching or Research)
      10. Other
12. City where you work?

__________________________________

1. In relation to working hours (outside your home), do you work as many hours a day?
2. Do you work on Saturdays? (regularly, at least 3 Saturdays a month)
   - 1. Yes
     2. No
3. Do you work nights, be they emergency calls or shifts? (at least 4 nights per month)
   - 1. Yes
     2. No
4. By month, what is the monthly estimate that you earn for your work in USD $ (adding all your income)

1. Do you agree that specialist doctors work 8 hours a day in public hospitals?
   - 1. Yes
     2. No
2. If the possibility of working 4 hours in a public hospital would be open, would you be willing to do so as long as any of the following conditions are met?
   - 1. If I would work but they should pay me the same as a full-time professional earns
     2. If I would work for a proportional salary that receives a full time
     3. I would not be willing to work in the public sector
     4. Other
3. What do you think is the main problem facing the Ecuadorian public health system (choose as many as you think necessary)?
4. On your usual day of work, what percentage of your time is spent filling out paperwork, paperwork and other administrative paperwork?
   1. 10%
   2. 10-20%
   3. 20-30%
   4. 30-40%
   5. 40-50%
   6. 50-60%
   7. 60-70%
   8. 70-80%
   9. 80%
   10. Not apply
5. Do you know what the Comprehensive Public Health Network is?
   - 1. Yes
     2. No
     3. Yes, but I do not know what is exactly
     4. I have heard talk about it, but I am not sure
6. What is the level of knowledge you have about the Organic Health Code (COES)?
   1. I have read it in parts, and I know something of its contents I have read it and I know its contents perfectly
   2. I have not read it and I know almost nothing of its content
   3. I have not read it, I've only heard of it and its contents
   4. I do not know what it is
7. . Do you agree that there is a regulation that regulates medical malpractice in Ecuador?
   - 1. Yes
     2. No
     3. Other
8. What percentage of doctors at the national level do you think do a bad job? (They are not updated, they are imprudent, they make mistakes, etc.)
   - 1. <10%
     2. 10-20%
     3. 30%
     4. 40%
     5. 50%
     6. 60%
     7. 70%
     8. 80%
     9. >80%
9. If you work in a public health center or hospital (MSP, IESS, ISFFA, ISSPOL) do you agree that there is an Administrative Manager and a Medical Director?
   - 1. No
     2. Other
     3. Yes, both
     4. Only medical director
     5. Only administrative manager
10. If you work in a public hospital (MSP, IESS, ISFFA, ISSPOL), have you ever been threatened with administrative sanctions so that you do not report a lack of medication, bad procedures and general administrative shortcomings? If you do not work in the public sector, mark not apply ***
    - 1. No
      2. Not Apply
      3. Yes
      4. Other
11. What is your perception of the usefulness of the National Table of Basic Drugs in Ecuador, considering that they are recommended by the WHO since the 1980s?
    - 1. Very useful
      2. Useful most times
      3. Little Useful in general
      4. Limit access to drugs
      5. Excludes recommended drugs
      6. Includes Drugs Little or Not Recommended
      7. It is a good tool, improves the rational use of medicines
      8. I do not know
12. What opinion do you have about the generic drugs that are marketed in Ecuador?
    - 1. They are all the same as the brand ones
      2. Most are of good quality
      3. Most are bad and ineffective
      4. Other
13. If you work in a public hospital, how many times do you (as a doctor) lack medicines or important medical supplies for the use of your patients? *** If you do not work in a public hospital, indicate "Not Apply"
    - 1. Usually
      2. Always
      3. Not apply
      4. Regularly
      5. Never
      6. Occasionally
      7. Rarely
14. If in the previous question, you answered yes, who do you think is responsible for this lack of medicines or supplies?
    - 1. From the Central Government for not giving resources
      2. From the Ministry of Health for not allocating resources
      3. From the Director or manager of the hospital for not planning properly
      4. Of the service managers for not planning adequately
      5. Other
15. In General, do you think public health care improved in the last 10 years? (There are more resources, better infrastructure, more medicines, better quality in public care)
    - 1. It is the same
      2. It is worst
      3. Yes, it improved on something
      4. Yes, it improved a lot
16. A) In your experience, which is or which are the most difficult medications to find in Ecuador, which are essential or necessary for your patients and they have had difficulty in acquiring them (difficult access) (you can put from 1 to 5).

B) In your experience, which is or which are the most difficult medications to find in Ecuador, which are essential or necessary for your patients and they have had difficulty in acquiring them (difficult access) (you can put from 1 to 5)

C) In your experience, which is or which are the most difficult medications to find in Ecuador, which are essential or necessary for your patients and they have had difficulty in acquiring them (difficult access) (you can put from 1 to 5)

D) In your experience, which is or which are the most difficult medications to find in Ecuador, which are essential or necessary for your patients and they have had difficulty in acquiring them (difficult access) (you can put from 1 to 5)

E) In your experience, which is or which are the most difficult medications to find in Ecuador, which are essential or necessary for your patients and they have had difficulty in acquiring them (difficult access) (you can put from 1 to 5)

1. The Ecuadorian State spends millions of dollars in medicines for catastrophic diseases, these are often extremely expensive and have very little impact on the course of the disease, if you would have to choose which patient to give those treatments and who not, what variables would you analyze?
   - 1. That the survival rate is greater than 50%
     2. That the total cost of pharmacological treatment does not exceed GDP per capita multiplied by three
     3. That the medication prolongs the life of the patient for at least 1 year
     4. Other
2. What is your opinion about foreign doctors who want to work in Ecuador?
   - 1. They are welcome as long as they pass the requirements of the professional qualification (Make rural, give an exam)
     2. They are welcome but should not occupy local places until it is shown that there are no local doctors interested
     3. They are not welcome
     4. All Doctors except Cuba Doctors are Welcome
3. What is your level of general satisfaction about Ecuadorian medical practice (0 very dissatisfied and 10 extremely satisfied)
   - 1. 0
     2. 1
     3. 2
     4. 3
     5. 4
     6. 5
     7. 6
     8. 7
     9. 8
     10. 9
     11. 10
4. Do you agree that all recent graduates of medical degrees at the national level take a professional qualification exam?
   - 1. Yes
     2. No
5. Do you think that specialists and general practitioners should "re-certify" every 5 years?
   - 1. Yes (CME) and exam
     2. Yes, only exam
     3. Yes (CME)
     4. No
     5. Others
6. How well or badly represented do you feel by the Ecuadorian Medical Federation (FME)?
   - 1. Terrible
     2. Very bad
     3. Bad
     4. Regular
     5. All right
     6. Very well
     7. Extremely well
7. In general, what do you think is the academic and research level of Ecuadorian scientific societies? (in general, knowing that scientific societies exist better than others)
   - 1. Terrible
     2. Very bad
     3. Bad
     4. Regular
     5. All right
     6. Very well
     7. Excellent
8. What activity do you think is most important within the competencies of the Ecuadorian Medical Federation (FME)?
   - 1. Academic activities
     2. Political Activities
     3. Trade union and union activities
     4. Partisan activities
     5. Other
9. If you were asked, would you be willing to attend a private number of patients (1-5) of the public health system for free in your private practice (only external consultation)?
   - 1. I would not be willing to treat any patient for free
     2. Yes, 1 per week
     3. Yes, up to 2 per week
     4. Yes, up to 3 per week
     5. Yes, up to 4 per week
     6. Yes, up to 5 per week
10. Do you think private health insurance harms you (to you as a health professional) in any way? ** If you do not work with private insurance, indicate "Not Apply".
    - 1. Yes
      2. No
      3. Not Apply
11. How would you rate health care within the public health sub-system of Ecuador (MSP) *** If you have no experience in the subject, have not used it or have not worked in it, say "Not apply".
    - 1. Not Apply
      2. Appalling
      3. Very bad
      4. Bad
      5. Regular
      6. Good
      7. Very good
      8. Excellent
12. How would you rate health care within the health sub-system of the Ecuadorian Institute of Social Security (IESS) *** If you have no experience in the subject, have not used it or have not worked in it, say "No apply"
    - 1. Not Apply
      2. Appalling
      3. Very bad
      4. Bad
      5. Regular
      6. Good
      7. Very good
      8. Excellent
      9. Outstanding
13. How would you rate health care within the health sub-system of the Social Security Institute of the Armed Forces of Ecuador (ISSFA)? *** If you have no experience in the subject, have not used it or have not worked in He, says "Not apply".
    - 1. Not Apply
      2. Appalling
      3. Very bad
      4. Bad
      5. Regular
      6. Good
      7. Very good
      8. Excellent
      9. Outstanding
14. How would you rate health care within the health sub-system of the Social Security Institute of the National Police (ISSPOL) *** If you have no experience in the subject, have not used it or have not worked in it, say "Not apply."
    - 1. Not Apply
      2. Appalling
      3. Very bad
      4. Bad
      5. Regular
      6. Good
      7. Very good
      8. Excellent
      9. Outstanding
15. How would you rate in general the operation of the National System of Public Health in Ecuador? (In General, the entire public sector)
    - 1. Not Apply
      2. Appalling
      3. Very bad
      4. Bad
      5. Regular
      6. Good
      7. Very good
      8. Excellent
      9. Outstanding
16. How would you rate in general the operation of the National System of Complementary System of Private Health in Ecuador?
    - 1. Not Apply
      2. Appalling
      3. Very bad
      4. Bad
      5. Regular
      6. Good
      7. Very good
      8. Excellent
      9. Outstanding
17. What is your general opinion about the level of quality of Ecuadorian medical attention in relation to the countries of the region?
    - 1. 1
      2. 2
      3. 3
      4. 4
      5. 5
      6. 6
      7. 7
      8. 8
      9. 9
      10. 10
18. Finally, what do you think is the Best Hospital of ALL THE PUBLIC HEALTH SYSTEM OF ECUADOR (Both public and private) in Ecuador, in terms of medical education, attention, cost-benefit, scientific and academic level of Ecuador.

________________________________________________________
